# The Impact of the COVID-19 Pandemic on the Perception of Health and Treatment-Related Issues among Patients with Phenylketonuria in Poland—The Results of a National Online Survey

**DOI:** 10.3390/ijerph18126399

**Published:** 2021-06-13

**Authors:** Dariusz Walkowiak, Bożena Mikołuć, Renata Mozrzymas, Łukasz Kałużny, Bożena Didycz, Dorota Korycińska-Chaaban, Michał Patalan, Joanna Jagłowska, Agnieszka Chrobot, Ewa Starostecka, Joanna Zarębska, Jarosław Walkowiak

**Affiliations:** 1 Department of Organization and Management in Health Care, Poznan University of Medical Sciences, Przybyszewskiego 39, 60-356 Poznan, Poland; 2 Department of Pediatrics, Rheumatology, Immunology and Metabolic Bone Diseases, Medical University of Bialystok, 15-274 Bialystok, Poland; bozenam@mp.pl; 3 Research and Development Center, Regional Specialist Hospital, 51-124 Wrocław, Poland; renata.mozrzymas@gmail.com; 4 Department of Pediatric Gastroenterology and Metabolic Diseases, Poznan University of Medical Sciences, 61-701 Poznan, Poland; lukasz@jerozolima.poznan.pl (Ł.K.); jarwalk@ump.edu.pl (J.W.); 5 Outpatient Metabolic Clinic, University Children’s Hospital, 30-663 Krakow, Poland; bozenadidycz@wp.pl; 6 PKU Polyclinic, Institute of Mother and Child, 01-211 Warsaw, Poland; chaaban@wp.pl; 7 Department of Pediatrics, Endocrinology, Diabetology, Metabolic Diseases and Cardiology, Pomeranian Medical University, 70-204 Szczecin, Poland; mpatalan@hotmail.com; 8 Department of Pediatrics, Hematology and Oncology, Medical University of Gdansk, 80-210 Gdansk, Poland; chabros_joanna@hotmail.com; 9 Voivodship Children Hospital, 85-667 Bydgoszcz, Poland; aga.chrobot@wp.pl; 10 The Regional Center of Rare Diseases, Polish Mother’s Memorial Hospital Research Institute, 93-338 Lodz, Poland; ewastarostecka@wp.pl; 11 Upper Silesian Child Health Centre, 40-752 Katowice, Poland; joanna.zarebska@gmail.com

**Keywords:** phenylketonuria, PKU, COVID-19 pandemic, compliance, remote medicine

## Abstract

There is agreement that the pandemic has affected the healthcare system and behaviour of patients. This study aims to identify problems encountered by patients with phenylketonuria (PKU) and their parents/caregivers during the six-week pandemic lockdown in Poland (15 March to 30 April 2020). To determine the factors that influenced health and treatment-related issues, as well as the respondents’ perception of the impact of the pandemic, study participants were asked to complete a non-validated online questionnaire comprising 31 questions (including 27 single-choice, two multiple-choice and two open-ended ones). A total of 571 patients or their parents completed the questionnaire, with 9.5% of respondents not performing any blood phenylalanine (Phe) test in the analysed period, 21.3% declaring a blood Phe increase, and 15.3% a decrease. Increased problems in contacting the doctor or dietitian were reported by 26.1% of subjects, whereas 39.3% of them felt restricted access to dietary products. Most (63.4%) participants were satisfied with remote contact with their PKU clinic. Better compliance was associated with higher odds of acceptance of remote contact and of reporting fewer problems with contacting the doctor, and with lower odds of missing Phe testing. Self-reported high stress was associated with higher odds of reporting the limited availability of low-Phe products and Phe-free formulas, as well as with increased Phe concentrations and non-PKU-related health problems. These patients also had poor dietary compliance and experienced more problems in contacting specialists. Health and treatment-related problems experienced during the pandemic lockdown were related to a higher intensity of stress in patient’s family and worse therapy compliance before the pandemic. Previous experience of remote visits resulted in a better perception of this method of contact. It seems that this form of communication should be popularized and improved to increase therapy effectiveness in case of different limitations in the future. Special attention should be paid to vulnerable patients who may be at extra risk when the provision of standard care is affected.

## 1. Introduction

When diagnosed by newborn screening and treated immediately, patients with phenylketonuria (PKU; OMIM 261600) essentially show normal development, although neuropsychological deficits and behavioral, emotional and social issues have been reported [1,2,3]. The social burden associated with having a chronic disorder and strict dietary treatment, especially for patients with severe forms of PKU, could affect their health-related quality of life (HrQoL) [4]. Cotugno et al. [5] found lower HrQoL for children and adolescents with PKU, while Landolt et al. [6] reported impaired positive emotional functioning in a group of PKU children and adolescents. Bilder et al. [4] also showed that rates of autism spectrum disorders and eating disorders are significantly higher in the adult PKU population compared to the general population, similarly for rates of depression and anxiety in the overall PKU population [7,8]. Jahja et al. [9] reported that adolescent and adult PKU patients demonstrated poorer social cognition and had poorer social skills than controls, with a tendency towards lower or delayed autonomy [10] and emotional difficulties related to maintaining a PKU diet [11]. Moreover, patients often feel social isolation and limits on socialisation [12], with many of them identified as having significant neurocognitive, mental health and general health problems [7,10,13,14]. An association between parent stress, anxiety, and depression in parents of PKU children has also been described [12,15,16,17,18]. Therefore, any additional external factor might aggravate the observed problems.

The coronavirus disease 2019 (COVID-19) pandemic has changed the management of many chronic diseases, including inborn errors of metabolism such as PKU. The first case of severe acute respiratory syndrome coronavirus 2 (SARS-CoV-2) infection in Poland was confirmed on 4 March 2020, after which the Polish government imposed various types of lockdown control measures, with all schools (including universities) across the country suspended; shortly afterwards, distance teaching began. In the meantime, companies and institutions limited their activities, often asking their employees to work from home and many layoffs began. During the first six-week pandemic lockdown in Poland (15 March to 30 April 2020), the situation in the health care system also changed. All scheduled appointments were cancelled. The patients were instructed to contact their doctors and other team members by phone or e-mail when needed. However, PKU laboratories were working in a routine way.

Although Poland was not affected by the first wave of COVID-19 pandemic to the same extent as other European countries, the measures taken by the Polish government to restrain the spread of COVID-19 could have had a significant impact on patients with PKU and their families. Apart from the circumstances related to the need to maintain the continuity of care for patients with PKU, a new, unrecognised factor appeared in connection with the pandemic: social isolation, that is, the inability to leave home and contact a doctor in person, as well as potential restrictions on the sale of various products, including food and medications. Therefore, the question arose of how these external circumstances related to the pandemic have affected the PKU treatment process and life of PKU patients in general.

The present study aimed to identify problems encountered by PKU patients and parents/caregivers during the six-week pandemic lockdown and determine the factors that influenced health and treatment-related issues in this group of patients.

## 2. Materials and Methods

The study was planned for all patients with PKU, regardless of age, under the care of one of the 10 specialist centres treating PKU patients in Poland. The inclusion criteria comprised: a diagnosis of PKU, implementation of dietary recommendations since the diagnosis, and systematic care in the metabolic clinic, including blood collection according to their age. All patients had full access to Phe-free formulas and special low-protein products that are covered by health insurance.

Invitations to participate in an online (Microsoft Forms), anonymous study were distributed to PKU patients and their caregivers by e-mails, text messages, during planned contact with PKU specialists, or by phone. Additionally, we asked for information about the survey to be placed on the websites of local PKU communities. To the best of our knowledge, information about the survey was also distributed by the patients on social networks and by phone. Patients over 16 years of age were asked to complete the survey by themselves, and for the younger ones, parents/caregivers were invited to do so. However, the parents/caregivers could fill out the survey for all ages. The data were collected between 15 June and 20 July 2020.

The questionnaire was designed by the research PKU team in cooperation with a specialist in public health. This non-validated questionnaire comprised 31 questions, including 27 single-choice, two multiple-choice and two open-ended questions. All questions concerned pandemic-related events and circumstances which, in our opinion, could have had a direct or indirect impact on health and treatment-related issues in patients with PKU. We also aimed to capture the patients’ perception of the impact of the pandemic. The questionnaire was designed using plain language and, wherever possible, specified response options using a five-point Likert scale graded from 1 (very dissatisfied/strongly disagree) to 5 (very satisfied/strongly agree) were used. The demographic part of the questionnaire consisted of questions about a patient’s age, gender, information—where applicable—about who was filling in the questionnaire on behalf of the patient (parents, other caregivers), place of residence understood in terms of the distance from a specialist hospital, and time and method of travel to the hospital. Respondents were also asked about a possible COVID-19 diagnosis or quarantine, as well as how and how often they remotely (using any means of indirect contact, e.g., phone calls or text messages; video chat; e-mail, Messenger, Whatsapp or Facebook contact) communicated with their doctors and/or dietitians both before and during the pandemic. We investigated possible problems in contacting specialists and the frequency of this contact. We also asked about the Phe levels in the patient’s blood before and during the pandemic and about problems maintaining their diet by the patients and in connection with possible problems with supply. Respondents were also asked about the perception of stress (on a scale graded from 1 to 10; 1 standing for low and 10 for high stress) they had experienced in connection with the pandemic. A series of questions concerned the assessment of remote contact with a specialist, the respondents’ willingness to continue this type of contact in the future, and the possibility of remotely treating PKU and other diseases. We also inquired about leaving home during the lockdown, about the activities in connection with which the patient would go outside, as well as contact with other PKU patients. In two open-ended questions, patients/their caregivers were encouraged to share a detailed assessment of remote visits and the impact of the pandemic (inability to leave home, increased responsibilities) on diet compliance. The questionnaire was preceded by written information about the purpose and method of the study, the researchers conducting the study were mentioned by name, and an e-mail address of the coordinator was provided.

Since the questionnaire was by definition anonymous, we analysed the repetitiveness of responses and unusual response times. The answers to open-ended questions, which were meant to eliminate the use of bots, were also analysed. The respondents were asked to check whether any other family member took part in the survey earlier.

The data collected in the questionnaires were verified, checked for completeness, quality and consistency and exported into the statistical package JASP (Version 0.12.2) and STATISTICA v. 13.3 (TIBCO, Palo Alto, CA, USA). The results are presented as descriptive statistics (means and standard deviations (SDs), ranges and percentages). The odds ratio (OR) was calculated to compare patients according to different characteristics based on specific feelings, behaviour or opinions in relation to other groups of patients. A 95% confidence interval (95% CI) was calculated to estimate the precision of the OR. The level of significance was set at *p* < 0.05.

## 3. Results

Out of the 575 completed questionnaires, 571 were qualified for further analysis and four questionnaires were excluded since the respondents were younger than 16. The mean age of the patients was 14.77 years (Table 1), with 296 (51.8%) females and 275 (48.2%) males. Almost three-quarters of the questionnaires were completed by the patients’ parents or other caregivers. Prior to the pandemic, nearly two-thirds of patients reported blood Phe levels within the recommended target for age; more than half stated that their results had not changed during the pandemic, although according to over 20% of respondents, Phe blood test results worsened in this period.

Most (53.4%) respondents declared that they used to contact their specialist doctor or dietitian by phone before the pandemic, whereas 10.1% contacted them using a video conference application or online messaging (Supplementary Table S1). During the pandemic, 58.3% of patients or their carers contacted their physician by phone, 20.0% by e-mail, and 13.5% by text message, with 31.9% of patients not feeling the need for such contact. A quarter (26.1%) of patients believed that problems with contact with their doctor and nutritionist increased, whereas 43.8% thought that they decreased. The number of patient contacts with their doctors or dietitians in the first six weeks of the pandemic in Poland ranged from 0 to 12.

Most (63.4%) of the surveyed patients/carers were satisfied with remote contact with a doctor and a dietitian, while only 4.4% were not satisfied (Supplementary Table S2). More than half (53.6%) of the respondents would like to use that form of contact in the future, while 19.0% of the respondents would not. Additionally, 40.8% of the respondents believed that in the case of PKU treatment, remote contact can replace direct contact with a specialist.

Limited access to special low-protein products and Phe-free formulas was reported by 39.3% and 20.7% of the respondents, respectively (Supplementary Table S3), with only 12.6% of patients considering that it was difficult to follow the diet in this period, whereas 42.2% were of the opposite opinion. Only 10.5% of patients during the pandemic spent time outdoors more often than before, in particular, for necessary housework, garden work and shopping. It should be noted, however, that as many as 24.0% of those surveyed reported practising sports in the period when it was prohibited.

According to the responses to the question “Did you use to contact your PKU specialist/dietitian by phone prior to the pandemic?” (Supplementary Table S1), the respondents were divided into three groups. The first group consisted of people who declared frequent telephone contact with their doctor (answer: regularly), the second group comprised those who declared that they contacted their doctor via telephone from time to time (answers: several times), and the remainder constituted the third group. The first and the second group were compared to the third group, calculating the OR of satisfaction with remote visits and willingness to use such contact in the future between groups. The ORs of being satisfied with remote contact with a PKU doctor for a person having regular/frequent contact in the past was higher than for those with no or occasional contact. Similarly, patients from the first and second groups were more interested in future remote contact (Table 2).

The respondents’ travel time to their doctor exceeding three hours was associated with a higher OR of their recognition of remote contact as a method of PKU treatment (Table 3).

The patients’ normal blood Phe level was associated with a higher OR of their satisfaction with remote contact as a method of PKU treatment (Table 4). The same patients had fewer problems contacting their doctor, lower odds of missing Phe testing and higher odds of being interested in future remote contact instead of in person.

High stress intensity (from 7 to 10) was associated with higher OR of reporting limited low-Phe product availability and limited Phe-free formula availability than lower feelings of stress (from 1 to 6). These patients also had poor dietary compliance, experienced more problems in contacting specialists, and a higher need to contact other PKU patients/parents. Self-reported high stress intensity was associated with higher OR of an increase in Phe concentrations and non-PKU-related health problems (Table 5).

## 4. Discussion

This study has identified health and treatment-related issues in PKU patients and their families resulting from the COVID-19 pandemic lockdown restrictions. The study findings are of important practical significance on many levels and in the short term may offer practical hints relevant in the case of another lockdown in the COVID-19 pandemic. Regarding the long-term perspective, our respondents’ experience of contact with the healthcare professionals contributes to their opinion on the usefulness of telemedicine in the future, after the end of the pandemic. Additionally, the study findings concern patients and their families, specialist doctors and dietitians who support patients in their fight against the disease, and the health care system as such, including the organisation of treatment. It is anticipated that our results could help to prepare healthcare providers for similar future events, to change the healthcare system, and to more adequately meet patients’ needs and expectations. Further actions would be crucial to sustain reasonable metabolic control as documented in the present study. To some degree, mental health problems in the population induced by the pandemic were predictable, as confirmed by the results of the latest mental health research [19,20,21,22]. There were also doubts about the health care system and its effectiveness in the face of information about new COVID-19 cases. Moreover, the changing situation could result in increased stress and non-compliance, with reduced physical activity causing a decrease in energy and macronutrient needs. Significant group of patients and their caregivers reported stress as an important problem, some of them also reporting shortages of low-Phe products. Moreover, even if the cause of anxiety or stress was not fully rational, this does not mean that stress did not affect the course of treatment. Nonetheless, various stress-related phenomena emerged in entire populations during the pandemic and the general atmosphere likely affected our respondents as well [23,24,25,26]. Our patients are routinely advised to follow an active lifestyle, and this recommendation could be of great importance, especially in terms of their psychological well-being.

The results of our study confirm previous findings regarding the need for psychological support to reduce levels of stress and anxiety during a pandemic for patients with rare diseases and their families, as well as for providing remote counselling to the extent that, due to the specificity of the disease, it is possible [27,28,29]. Similar to the findings concerning patients with cystic fibrosis (CF) [29], our patients also reported changes in daily routine during self-isolation; this was mostly unintentional non-adherence to treatment for CF, whereas, in our study, most respondents’ self-reported adherence was better, though some reported poor adherence or complete non-adherence to their diet. As it has been shown earlier [30], compliance deteriorates with the patient’s age, though this study suggests that compliance may also be influenced by external factors. Regardless of the readiness of the healthcare system, the application of telemedicine in the new COVID-19 reality is unquestionable. Special emphasis should be put in the future on video consulting and everyone should contribute to the increase of its use. A wide-ranging information campaign regarding the possibility of remote visits to doctors of various specialties was launched in March 2020, with the pandemic accelerating the implementation and popularisation of remote visits among patients in different countries and suffering from different diseases [20,31,32,33]. The possibility of using remote visits in the treatment of rare diseases [34,35], paediatric treatment [36] or chronic diseases [37,38] had been discussed previously. The applicability of such treatment in patients with PKU has remained outside the mainstream, but the pandemic has shown that, to some extent, remote visits are possible.

Opinions on remote visits for patients with PKU and their parents are closely related to their previous experience with this form of treatment. Since many patients do not have such experience, opinions about the visits also varied. It seems that people in Poland do not need to be convinced to use video calls as a form of communication [39], but there is a need to enable them to use this tool, not only for private purposes but also for patient–doctor communication. This requires adjustments on the part of the health service, both in terms of the willingness of teams participating in the treatment of patients with PKU to engage in remote medicine but also in terms of changes in the infrastructure of the health care system. Future lockdowns being a possibility, there is no other option but to familiarise patients with remote visits, especially as they are likely to become the only possible type of contact soon. For patients who have doubts as to the effectiveness of remote treatment, the healthcare system should make the first move to initiate such visits.

There are some limitations to this study. Firstly, we used a non-validated questionnaire. Secondly, we referred to only a six-week lockdown. One could have expected more disparities if we had assessed a longer period with a possibly worse effect for the most vulnerable. The strength of the present study is its sample size, with a high response rate (definitely exceeding 50%).

In summary, pandemic-related self-reported stress in many patients with PKU or/and their caregivers was very high and more frequently resulted in lower treatment compliance. These patients also more often reported problems contacting a specialist, which, taking into account their more frequent Phe results over the therapeutic range, may be an attempt at self-justification. It is necessary to quickly identify patients from that group and implement psychological support, as well as to try to reimplement the diet and compliance regime. One-third of patients did not have contact with a doctor or nutritionist during the pandemic, with 1 in 10 patients not performing blood tests during the lockdown and one in five patient’s Phe results worsened. For at least 30% of patients, this urgently requires the restoration of the pre-pandemic metabolic control. The results of our study suggest a significant number of people experienced stress; however, considering the nature of the survey and the possible bias resulting from the fact that the group was not fully representative, the situation in the entire population of PKU patients may be even worse than our results indicate [40].

As we can see, our patients accept the solutions they are familiar with; a possibly long future period of non-existent or limited personal contact requires the preparation of patients for forms of therapy that enable supervision and support in maintaining metabolic control. Additionally, for patients with a long travel time to the hospital, it seems appropriate to consider maintaining remote visits to some extent even after the coronavirus threat is over, subsequently offering such an option to patients living closer to PKU treatment centres provided they can engage well in the methods used. The healthcare providers should define the most vulnerable patients at the highest risk of losing proper control. It is important when establishing remote clinics in the future to bear in mind that this may actually intensify social disparities. When choosing a treatment method, it is crucial to define individual needs rather than adopt the same approach for everyone. More vulnerable patients may need more face-to-face contact.

## 5. Conclusions

Health and treatment-related problems experienced during the pandemic lockdown were related to a higher intensity of stress in patients’ families and worse therapy compliance before the pandemic. Previous experience of remote visits resulted in a better perception of this method of contact. The popularisation and improvement of this form of communication is warranted to increase the effectiveness of therapy in case of different limitations in the future, such as a new lockdown. It is also a promising attitude for future PKU treatment regardless of the pandemic, especially in patients experiencing problems with visiting a PKU centre.

## Figures and Tables

**Table 1:** Respondents’ characteristics (N = 571).

Characteristics	n/N (%)
Number of patients	571
Patient gender	
Female	296 (51.8)
Male	275 (48.2)
Mean age (SD)	14.77 (12.6)
Age range	0.17–53
Missing age data	4 (0.7)
Number of patients >26 years under parents’ supervision	42 (7.4)
Questionnaire filled in by parents	424 (74.3)
Questionnaire filled in by patients	147 (25.7)
Quarantined	11 (1.9)
Self-reported Phe levels before the pandemic	
As recommended	363 (63.6)
Slightly too high	174 (30.5)
Far too high	34 (5.9)
Self-reported Phe levels during the pandemic lockdown	
Increased considerably	27 (4.7)
Increased slightly	95 (16.6)
Remained the same	302 (52.9)
Decreased slightly	75 (13.1)
Decreased considerably	18 (3.2)
No tests in lockdown period	54 (9.5)
Distance between household and the clinic	
In our city	102 (17.9)
Less than 50 km	112 (19.6)
50–100 km	128 (22.4)
More than 100 km	229 (40.1)
Mode of travel to clinic	
Own car	453 (79.3)
Rented car	15 (2.6)
City transport	45 (7.9)
Train or intercity bus	45 (7.9)
Several trains or buses	13 (2.3)
Travel time to doctor	
Less than 0.5 h	105 (18.4)
0.5–1 h	153 (26.8)
1–2 h	174 (30.5)
2–3 h	91 (15.9)
More than 3 h	48 (8.4)

N—number of all respondents, n—number of a given answer, SD—standard deviation, Phe—phenylalanine.

**Table 2:** The impact of contact frequency with a doctor before the pandemic on satisfaction with remote visits.

	Phone Contact Frequency	
	Group 1 (Patients with Regular Phone Contact with PKU Doctor/Dietitian before Pandemic) vs. Group 3 (Patients with Occasional/No Contact)	Group 2 (Patients with Several Phone Contacts with PKU Doctor/Dietitian before Pandemic) vs. Group 3 (Patients with Occasional/No Contact)
	Were you satisfied with the remote contact?	
OR	22.47	8.30
95% CI	12.57–40.14	5.34–12.90
*p*	<0.0001	<0.0001
	Would you like to have remote contact in the future?	
OR	2.35	1.98
95% CI	1.54–3.59	1.34–2.93
*p*	<0.0001	0.0003

PKU—phenylketonuria, OR—odds ratio, CI—confidence interval.

**Table 3:** The impact of travel time for a medical visit before the pandemic on satisfaction with remote visits.

	Travel Time to the Centre	
	How Long Does It Take to Get to Your Doctor?	
	>3 h to the Doctor vs. All Others	>2 h vs. <1 h
OR	2.17	1.77
95% CI	1.19–3.96	1.17–2.69
*p*	0.0057	0.0037

OR—odds ratio, CI—confidence interval.

**Table 4:** Self-reported Phe levels (normal vs. abnormal) before the pandemic and the perception of remote visits, availability of specialists and the lack of control Phe tests during the pandemic.

	Self-Reported Phe Levels (Normal vs. Abnormal)			
	Satisfaction with Remote Contact	Problems in Contacting a Specialist	No Tests during the Pandemic	Positive Opinion on Remote Contact as a Means of Treating PKU
OR	1.56	0.59	0.42	1.68
95% CI	1.10–2.22	0.40–0.87	0.24–0.76	1.10–2.56
*p*	0.0063	0.0034	0.0020	0.0080

PKU—phenylketonuria, OR—odds ratio, CI—confidence interval.

**Table 5:** Comparison of patients’ frequency of reporting high stress vs. low-intermediate stress intensity depending on social interactions.

	Social Interactions (Declarations)				
	Limited Low-Phe Product Availability	Limited Phe-Free Formula Availability	Worse Dietary Compliance	Need to Contact Other Parents/Patients	Problems in Contacting a Specialist
OR	2.00	1.87	2.03	1.97	1.55
95% CI	1.42–2.84	1.24–2.64	1.24–3.35	1.22–3.19	1.06–0.27
*p*	<0.0001	<0.0001	<0.0001	0.0028	0.0016
	**Social Interactions (Declaration/Report)**				
	**an Increase of Phe Concentrations**			**Other Than PKU-Related Health Problems**	
OR	1.95			3.67	
95% CI	1.23–3.10			1.75–7.69	
*p*	0.0023			0.0003	

Phe—phenylalanine, PKU—phenylketonuria, OR—odds ratio, CI—confidence interval.

## Data Availability

The datasets used and/or analyzed in the current study are available from the corresponding author on reasonable request.

## Supplementary Materials

The following are available online at https://www.mdpi.com/article/10.3390/ijerph18126399/s1, Table S1: Characteristics of contacts with a PKU specialist doctor and dietitian (N = 571), Table S2: Respondents’ opinions on remote contact in the treatment of PKU (N = 571), Table S3: Respondents’ opinions on access to special food, diet, activities and stress during the pandemic (N = 571).

## Abbreviations

PKU: phenylketonuria: Phe: phenylalanine, PAH: phenylalanine hydroxylase.
