# Supplementary material for: The Impact of the COVID-19 Pandemic on the Perception of Health and Treatment-Related Issues among Patients with Phenylketonuria in Poland—The Results of a National Online Survey
Source: Int J Environ Res Public Health. 2021 Jun 13;18(12):6399. doi: 10.3390/ijerph18126399 (PMC8296241; doi:10.3390/ijerph18126399)
Supplement: Supplementary file 1 [file ijerph-18-06399-s001.zip › ijerph-1182710-supplementary.pdf]

International Journal of
Environmental Research and Public Health

Supplementary Table 1. Characteristics of contacts with a PKU specialist doctor and dietitian (N=571).

Characteristics	n/N (%)
Did you use to contact your PKU specialist/dietitian by phone prior to the pandemic?	
Yes, regularly	138 (24.2)
Yes, several times	167 (29.2)
Maybe once	56 (9.8)
I do not remember	23 (4.0)
I did not feel such a need	178 (31.2)
There was no such possibility	9 (1.6)
Before the pandemic, did you use to contact your PKU specialist/dietitian via Skype, videoconference or through social media?	
Yes, regularly	27 (4.7)
Yes, several times	31 (5.4)
Maybe once	14 (2.5)
I do not remember	25 (4.4)
I did not feel such a need	333 (58.3)
There was no such possibility	141 (24.7)
How did you contact your doctor or dietitian during the pandemic?	
Phone	333 (58.3)
Videochat	2 (0.4)
Messenger & WhatsApp	16 (2.8)
Facebook	5 (0.9)
Text message	77 (13.5)
E-mail	114 (20.0)
I did not feel such a need	182 (31.9)
There was no such possibility	10 (1.8)
Have pandemic restrictions changed the difficulty to communicate with your specialist doctor/dietitian?	
Increased considerably	23 (4.0)
Increased slightly	126 (22.1)
Remained similar	172 (30.1)
Decreased slightly	90 (15.8)
Decreased considerably	160 (28.0)
Number of contacts with a doctor	
Mean (SD)	1.7 ±2.1
Range	0–12
Missing data	5

PKU – phenylketonuria, N – number of all respondents, n – number of a given answer, SD – standard deviation,

Supplementary Table 2. Respondents' opinions on remote contact in the treatment of PKU (N=571).

**Characteristics**	**n/N (%)**
Were you satisfied with the remote contact?	
Definitely	199 (34.9)
Yes	163 (28.5)
I do not know	184 (32.2)
No	17 (3.0)
Definitely not	8 (1.4)
Would you like to have remote contact in the future?	
Definitely	137 (24.0)
Yes	169 (29.6)
I do not know	157 (27.5)
No	82 (14.4)
Definitely not	26 (4.6)
In the case of PKU, can a remote visit replace direct contact with a specialist?	
Definitely	48 (8.4)
Yes	185 (32.4)
I do not know	61 (10.7)
No	196 (34.3)
Definitely not	81 (14.2)
In the case of other diseases, can you imagine remote contact with the doctor?	
Definitely	40 (7.0)
Yes	116 (20.3)
I do not know	78 (13.7)
No	245 (42.9)
Definitely not	92 (16.1)

PKU – phenylketonuria, N – number of all respondents, n – number of a given answer,

Supplementary Table 3. Respondents' opinions on access to special food, diet, activities and stress during the pandemic (N=571).

What was your access to special low phenylalanine products during the pandemic?	**n/N (%)**
Definitely worse	34 (6.0)
Worse	190 (33.3)
The same as before the pandemic	342 (59.9)
Better	2 (0.3)
Definitely better	3 (0.5)
What was your access to phenylalanine-free formulas during the pandemic?	
Definitely worse	18 (3.2)
Worse	100 (17.5)
The same as before the pandemic	445 (79.3)
Better	4 (0.7)
Definitely better	4 (0.7)
Was it difficult to stick to the diet during the pandemic?	
Definitely more difficult	9 (1.6)
More difficult	63 (11.0)
The same as before the pandemic	258 (45.2)
Easier	136 (23.8)
Definitely easier	105 (18.4)
During the pandemic and its associated mobility restrictions, have there been any other health problems that may have affected your Phe results?	
Definitely	28 (4.9)
Yes	22 (3.9)
I do not know	30 (5.2)
No	164 (28.7)
Definitely not	327 (57.3)
What was the intensity of stress* in the patient's family in connection with the pandemic?	
1	88 (15.4)
2	36 (6.3)
3	52 (9.1)
4	52 (9.1)
5	85 (14.9)
6	43 (7.5)
7	72 (12.6)
8	67 (11.7)
9	35 (6.1)
10	41 (7.2)
During the pandemic, have you contacted other PKU patients, their parents or caregivers more often than before for information about the diet?	
Yes	493 (86.3)
No	78 (13.7)
Frequency of PKU patient's outdoor activities/walks/play between 13 March and 30 April	
Increased considerably	18 (3.2)
Increased slightly	44 (7.7)
Remained similar	207 (36.2)
Decreased slightly	176 (30.8)
Decreased considerably	126 (22.1)
Under what circumstances did a person with PKU leave home between 13 March and 30 April?	
Did not leave home at all	49 (8.6)
Walks with a dog	162 (28.4)
Necessary household duties	173 (30.3)
Grocery shopping	134 (23.5)
Shopping in a pharmacy	78 (13.7)
Going out to work	61 (10.7)
Visits to a doctor	35 (6.1)
Gardening	169 (29.6)
Sports	137 (24.0)

N – number of all respondents, n – number of a given answer, PKU – phenylketonuria,
*1 being low and 10 high stress
